# Multidrug resistant bacteria are sensitive to *Euphorbia prostrata* and six others Cameroonian medicinal plants extracts

**DOI:** 10.1186/s13104-017-2665-y

**Published:** 2017-07-25

**Authors:** Igor K. Voukeng, Veronique P. Beng, Victor Kuete

**Affiliations:** 1 Department of Biochemistry, Faculty of Science, University of Dschang, Dschang, Cameroon; 2 Department of Biochemistry, Faculty of Science, University of Yaounde I, Yaounde, Cameroon

**Keywords:** Antimicrobial, Cameroon, *Euphorbia prostrata*, Medicinal plant, Multidrug resistance

## Abstract

**Background:**

Multidrug resistant (MDR) bacteria are responsible for therapeutic failure and there is an urgent need for novels compounds efficient on them.

**Methods:**

Eleven methanol extracts from seven Cameroonian medicinal plants were tested for their antibacterial activity using broth micro-dilution method against 36 MDR bacterial strains including *Escherichia coli*, *Enterobacter aerogenes*, *Enterobacter cloacae*, *Klebsiella pneumoniae*, *Providencia stuartii*, *Pseudomonas aeruginosa* and *Staphylococcus aureus*.

**Results:**

*Euphorbia prostrata* extract was found active against all the 36 tested bacteria including Gram-negative phenotypes over-expressing efflux pumps such as *P. aeruginosa* PA124, *E. aerogenes* CM64 and *E. coli* AG102. *E. prostrata* had minimal inhibitory concentrations values between 128 and 256 µg/mL on 55.55% of the studied microorganisms. Other plants extract displayed selective antibacterial activity.

**Conclusions:**

Results obtained in this study highlight the antibacterial potential of the tested plants and the possible use of *E. prostrata* to combat bacterial infections including MDR phenotypes.

## Background

Bacterial multidrug-resistance is the ability of bacteria to grow in the presence of antibiotics at concentrations that were previously inhibitory. Treating infections caused by multidrug-resistant (MDR) bacteria is a challenge more and more difficult to solve within hospital units [1]. Although the resistance of bacteria to antibiotics is a natural adaptation phenomenon, the rapid emergence of MDR phenotypes is mainly due to misuse of antibiotics, which increases the selection pressure and favors the appearance MDR microorganisms [2]. In hospitals, patients infected by these bacteria stay for long time, which impacts on the cost of treatment. Faced with this crisis, it is important to develop new antibacterial molecules effective vis-à-vis of MDR bacteria and medicinal plants offer a suitable alternative. According to WHO, 80% of world population uses medicinal plants for their health needs; antibacterial potential against the multidrug resistant phenotypes of many of them like *Afromomum citratum*, *Afromomum melegueta*, *Imperata cylindricum*, *Cinnamomum zeylanicum*, *Dioscorea bulbifera*, *Dorstenia psilurus* has already been demonstrated [3, 4]. In order to contribute to the discovery of active substances from medicinal plants, this study was designed to assess the antibacterial potential of different parts of *Aloe buettneri* A. Berger (Asphodelaceae), *Alchornea floribunda* Müll. Arg. (Euphorbiaceae), *Crinum purpurascens* Herb. (Amaryllidaceae), *Euphorbia prostrata* Ait. (Euphorbiaceae), *Markhamia tomentosa* K. Schum. (Bignoniaceae), *Viscum album* L. (Loranthaceae) and *Rauwolfia macrophylla* Ruiz & Pav. (Apocynaceae) against MDR Gram-positive and Gram-negative phenotypes.

## Methods

### Plant material and sample preparation

Different part of the investigated plants including leaves, stem, stem bark, bark or whole plant (Table 1) were harvested in different regions of Cameroon. The plants were then identified at Cameroon National Herbarium where the voucher specimens are available (Table 1). The dry powders (200 g) of each part of plants were soaked in methanol for 48 h; the filtrates obtained after filtration paper through Whatman No. 1 were concentrated under reduced pressure and the obtained extracts were kept at 4 °C for further biological tests.

**Table 1:** Information on studied species

Plant species, (voucher number)/family	Traditional use	Part used traditionally	Potential active constituents	Previously screened activity
*Aloe buettneri* A. Berger (59062/HNC)/Asphodelaceae	Gastro-intestinal infections, chronic wounds, cutaneous infections, inflammations, gastric ulcers, chronic skin ulcer, cough, dysmenorrhea, food poisoning, difficult delivery, dysentery, general stomachaches, lumbar painregulation of menstrual cycle, functional infertility [18–20]	Leaves	–	Anti-ulcer, anti-inflammatory, ovarian steroidogenesis effect, sub-acute toxicity, Analgesic effect, Antipyretic activity [18–20]
*Alchornea floribunda* Müll. Arg. (4595/HNC)/Euphorbiaceae	Hepatitis, wounds, ringworm, eczema, pains in the heart, antidote to poison, urinary, respiratory and intestinal disorders, aphrodisiac [21, 22]	Leaves, bark, root	Cathechin, epicathechin, taxifolin, 5α-stimastane-3,6-dione, 3β-hydroxyl-5α-stigmastane-24-ene, 5α-stigmastane-23-ene-3,6-dione [21, 23]	Antibacterial, anti-inflammatory, antioxidant, antiprotozoal, cytotoxicity [15, 21–23]
*Crinum purpurascens* Herb. (40058/HNC)/Amaryllidaceae	Pneumonia, ovarian problems, hernia, wounds, dysentery, microbial infection, aphrodisiac, snake bite [24]	Leaves, bulbs	Hippadine, pratorimine, β-d-glucopyranoside sitosterol [25]	Antibacterial [25]
*Euphorbia prostrata* Ait. (33585/HNC)/Euphorbiaceae	Infertility, menstrual pain, dysentery, typhoid fever [12]	Leaves, whole plant	Prostratins A, B and C, rugosins A, D, E and G, quercetin 3-*O*-β-sambubioside, euphorbins G and H, tellimagradin I and II, kaempferol, cosmosiin, rhamnetin-3-galactoside, quercetin, β-amyrine acetate, β-sitosterol, campesterol, stigmasterol and cholesterol [26, 27]	Antibacterial, antifungal, bleeding haemorrhoids [12, 28, 29]
*Markhamia tomentosa* K. Schum. (1974/SRF-Cam)/Bignoniaceae	Edema, cancer, gout, scrotal elephantiasis, pulmonary troubles, general body pain [30]	Leaves	β-sitosterol, β-sitosterol-3-*O*-β-d-glucopyranoside, dehydroiso-α-lapachone, 2-acetylnaphtho[2,3-b]furan-4,9-dione, pomolic acid, 2-acetyl-6-methoxynaphtho[2,3-b]furan-4,9-dione, β-lapachone, tormentic acid, oleanolic acid, palustrine [31, 32]	Antiprotozoal, antihelmintic, antibacterial, antifungal, antiviral, analgesic, anti-inflammatory antioxidant, anti-tumor [31–33]
*Viscum album* L. (2974/HNC)/Loranthaceae	Atherosclerosis, hypertension, cancer, headache, dizziness, palpitation [34, 35]	Leaves	Coniferin, syringin, eleutheroside E, syringaresinol-*O*-glucoside, ligalbumosides A–E, alangilignoside C, kalopanaxin D, β-amyrin acetate, β-amyrin, lupeol, lupeol acetate oleanolic acid, betulinic acid, stigmasterol, β-sitosterol, *trans*-α-bergamotene, *trans*-β-farnesene, loliolide, vomifoliol, 5,7-dimethoxy-flavanone-4′-*O*-glucoside, 2′-hydroxy-4′,6′-dimethoxychalcone-4-*O*-glucoside, 5,7-dimethoxyflavanone-4′-*O*-[2′′-*O*-(5′′′-*O*-*trans*-cinnamoyl)-apiosyl]-glucoside), 2′-hydroxy-4′,6′-dimethoxychalcone-4-*O*-[2′′-*O*-(5′′′-*O*-*trans*-cinnamoyl)-apiosyl]-glucoside, 2′-hydroxy-3,4′,6′-trimethoxychalcone-4-*O*-glucoside, 2′-hydroxy-4′,6′-dimethoxychalcone-4-*O*-[apiosyl(1 → 2)]-glucoside, 5,7-dimethoxyflavanone-4′-*O*-[apiosyl-(1 → 2)]-glucoside, (2*S*)-3′,5,7-trimethoxyflavanone-4′-*O*-glucoside, 4′-*O*-[β-apiosyl(1 → 2)]-β-glucosyl]-5-hydroxy-7-*O*-sinapylflavanone, 3-(4-acetoxy-3,5-dimethoxy)-phenyl-2*E*-propenyl-β-glucoside, 3-(4-hydroxy-3,5-dimethoxy)-phenyl-2*E*-propenyl-β-glucoside, 4′,5-dimethoxy-7-hydroxy flavanone, 5,7-dimethoxy-4′-hydroxy flavanone [36]	Antihypertensive, cytotoxicity, vascular effects, antioxidant, antibacterial [34, 35, 37, 38]
*Rauwolfia macrophylla* Ruiz & Pav. (1697/SRFK)/Apocynaceae	Measles or itching rash, fever, swellings, rheumatism, hepatitis, pneumonia, abdominal pain, cough and toothache, headache, insomnia and palpitation of the heart, abscess, roundworm, tapeworm, hypertension, epilepsy, eye diseases, venereal diseases [39]	Leaves, bark, roots	Reserpine, rescinnamine, ajmaline, methyl reserpate, normacusine B, suaveoline, serpentine, perakine, vomilenine, peraksine, dihydroperaksine, norajmaline, ajmalicinine, ajmalicine, geissoschizol, pleiocarpamine, α-yohimbine, alloyohimbine, yohimbine [40–43]	Antimycobacterial and Antibacterial activity, antioxidant [13, 44, 45]

### Phytochemical screening

The presence of compounds belonging to different classes of secondary metabolites was determined according to described methods [5].

### Chemicals for antimicrobial assay

The reference antibiotic (RA) used against bacteria were chloramphenicol and ciprofloxacin (Sigma-Aldrich, St. Quentin Fallavier, France) meanwhile the bacterial growth indicator was *p*-iodonitrotetrazolium chloride ≥ 97% (INT, Sigma-Aldrich).

### Microbial strains and culture media

Microorganisms used in this study included 36 multidrug-resistant strains of Gram-negative belonging to *Escherichia coli*, *Enterobacter aerogenes*, *Enterobacter cloacae*, *Klebsiella pneumoniae*, *Providencia stuartii*, *Pseudomonas aeruginosa* species and Gram-positive bacteria belonging to and *Staphylococcus aureus* species (Table 3). Their bacterial features were previous reported [6]. Mueller–Hinton Agar (Sigma) was used to activate the microorganisms whilst Mueller Hinton broth (MHB; Sigma) was used for antibacterial assays.

### INT colorimetric assay for MIC and MBC determinations

The minimal inhibitory concentrations (MIC) and minimal bactericidal concentrations (MBC) of different plant extracts were determined by a by the rapid INT colorimetric assay according to described methods [3]. Chloramphenicol and ciprofloxacin were the reference drugs for Gram-negative and Gram-positive respectively. Each plant extract was dissolved in a 5% DMSO solution; an aliquot of 100 μL was added to the wells of a microplate containing 100 μL of MHB; then serial dilution was performed. A bacterial suspension corresponding to 0.5 McFarland scale was prepared and diluted 100 times in the sterile MHB. Afterwards, 100 μL of the bacterial suspension was added to all wells and the plate was incubated at 37 °C for 18 h. Bacterial growth was detected by adding 40 μL of INT (0.2 mg/mL) and the appearance of pink color indicated bacterial growth; the lowest concentration of the extract where no color change was observed was recorded as MIC.

For the determination of MBCs, we used new 96-well plates containing 150 μL of MHB in which we added an aliquot of 50 μL from the wells corresponding to MIC as well as upper concentrations. Those microplates were incubated at 37 °C for 48 h and revelation was done as mentioned above and the lowest concentration indicating the absence of bacterial growth was considered as MBC. Each of the experiments was carried out in triplicate.

## Results

The classes of secondary metabolites present in extracts of different parts of plants were detected and results are summarized in Table 2. Alkaloids, triterpenes, sterols, flavonoids, polyphenols and saponins were screened in all plant extracts. Other classes of compounds were selectively distributed.

**Table 2:** Phytochemical composition and extraction yield of studied parts of plant

Plant name	Used part and extraction yield	Alkaloids	Triterpenes	Sterols	Flavonoids	Polyphenols	Saponins
*A. buettneri*	Whole plant (8.15%)	+	+	−	−	−	+
*A. floribunda*	Leaves (4.36%)	−	+	+	−	−	+
	Stem-bark (2.88%)	+	+	−	−	+	+
*C. purpurascens*	Leaves (6.85%)	+	+	+	−	+	+
*E. prostrata*	Whole plant (9.14%)	−	−	+	+	+	+
*M. tomentosa*	Leaves (8.39%)	−	+	+	−	+	+
	Bark (5.31%)	−	+	−	−	+	+
*V. album*	Leaves (4.97%)	+	+	+	+	+	−
	Stem (14.85%)	+	+	+	+	+	−
*R. macrophylla*	Leaves (11.58%)	+	+	−	−	−	+
	Bark (17.26%)	+	+	−	−	−	+

The results of antibacterial tests are summarized in Table 3. It appears that the plant extract from *E. prostrata* was active against all tested bacterial strains (36/36) with MIC between 128 and 256 µg/mL on 55.55% (20/36) of the studied microorganisms. The methanol extracts of leaves and stems of *V. album* as well as other plant extracts displayed selective activities on MDR Gram-negative as well as on methicillin-resistant *S. aureus* (MRSA) strains. Leaves extract of *R. macrophylla* were active against 83.33% (30/36) of the tested bacteria while only 3/36 (8.33%) of the studied bacteria were sensitive to the bark extract. Leaves extract of *M. tomentosa* was active on 75% (27/36) of bacterial strains while the extracts of bark were active only on 30.55% (11/36). The methanol extract from the leaves of *C. purpurascens* showed activity against all MRSA strains and 22/29 Gram-negative bacteria tested; except bacteria belonging to *P. aeruginosa* species, all other bacterial species studied herein were susceptible to *C. purpurascens* leaves extract. *E. coli*, *P. aeruginosa*, *E. aerogenes* and *P. stuartii* strains were mostly resistant to the action of the extract of leaves of *A. floribunda*. The extract of *A. buettneri* leaves had weak activity, its inhibitory effects being observed against 3/36 (8.33%) bacterial strains.

**Table 3:** Antibacterial activity of studied parts of plant (MIC and MBC are in μg/mL)

Bacterial strains	*Alchornea floribunda*		*Aloe buettneri* (leaves)	*Markhamia tomentosa*		*Euphorbia prostrata* (whole plant)	*Viscum album*		*Crinum purpurascens* (leaves)	*Rauwolfia macrophylla*		RA
	Leaves	Stem bark		Leaves	Bark		Leaves	Stem		Leaves	Bark	Chloramphenicol
*E. coli*												
ATCC8739	–	–	–	–	–	256 (−)	512 (−)	256 (−)	128 (1024)	256	–	2 (128)
ATCC10536	–	1024 (−)	–	512 (−)	–	256 (−)	256 (−)	512 (−)	256 (−)	256 (−)	–	<2 (64)
AG100	–	–	–	128 (−)	256 (−)	128 (512)	512 (−)	512 (−)	–	512 (−)	1024 (−)	8 (128)
AG100A	–	–	512 (−)	128 (−)	128 (512)	256 (256)	–	–	256 (−)	–	–	<2 (128)
AG100A_TET_	–	–	–	512 (−)	–	512 (−)	1024 (−)	256 (−)	1024 (−)	256 −)	–	32 (−)
AG102	1024 (−)	512 (−)	–	1024 (−)	–	256 (−)	512 (−)	512 (−)	512 (−)	256 (−)	–	64 (−)
MC4100	–	1024 (−)	–	256 (−)	–	512 (−)	128 (−)	512 (−)	512 (−)	–	–	16 (−)
W311O	–	256 (−)	–	512 (−)	–	1024 (−)	1024 (−)	256 (−)	256 (−)	256 (−)	128 (−)	2 (−)
*P. aeruginosa*												
PA 01	–	1024 (−)	–	256 (−)	–	256 (−)	256 (−)	256 (−)	–	128 (−)	–	32 (−)
PA 124	–	–	–	–	–	256 (−)	–	–	–	–	–	128 (−)
*E. aerogenes*												
ATCC13048	1024 (−)	512 (−)	–	1024 (−)	–	512 (−)	–	256 (−)	–	256 (−)	–	4 (32)
EA-CM64	–	–	–	1024 (−)	1024 (−)	256 (−)	–	512 (−)	1024 (−)	1024 (−)	1024 (−)	256 (−)
EA3	–	256 (−)	–	–	1024 (−)	128 (−)	–	512 (−)	256 (−)	512 (−)	–	256 (−)
EA27	512 (−)	512 (−)	256 (−)	256 (−)	1024 (−)	256 (−)	256 (−)	1024 (−)	256 (−)	256 (−)	–	32 (−)
EA289	–	512 (−)	–	512 (−)	1024 (−)	512 (−)	1024 (−)	1024 (−)	1024 (−)	256 (−)	–	64 (−)
EA298	–	256 (−)	–	512 (−)	1024 (−)	512 (−)	1024 (−)	–	–	256 (−)	–	128
*P. stuartii*												
NEA16	512 (−)	256 (−)	–	–	–	256 (−)	1024 (−)	1024 (−)	–	256 (−)	–	32 (256)
ATCC29916	–	1024 (−)	–	–	–	512 (−)	256 (−)	1024 (−)	512 (−)	512 (−)	–	16 (256)
PS2636	–	1024 (−)	–	–	–	256 (−)	256 (−)	1024 (−)	512 (−)	512 (−)	–	16 (256)
PS299645	–	512 (−)	–	–	–	256 (−)	512 (−)	1024 (−)	512 (−)	256 (−)	–	64 (−)
*K. pneumoniae*												
ATCC11296	512 (−)	1024 (−)	–	512 (−)	256 (−)	256 (−)	512 (−)	512 (−)	512 (−)	1024 (−)	–	8 (256)
KP55	256 (−)	512 (−)	512 (−)	512 (−)	256 (−)	256 (−)	256 (−)	1024 (−)	256 (−)	512 (−)	–	32 (256)
KP63	–	–	–	1024 (−)	1024 (−)	512 (1024)	512 (−)	1024 (−)	512 (−)	512 (−)	–	32 (−)
K24	512 (−)	1024 (−)	–	512 (−)	–	256 (−)	512 (−)	512 (−)	512 (−)	1024 (−)	–	64 (256)
K2	512 (−)	512 (−)	–	512 (−)	128 (−)	1024 (−)	512 (−)	1024 (−)	512 (−)	512 (−)	–	8 (256)
*E. cloacae*												
ECCI69	1024 (−)	1024 (−)	512 (−)	1024 (−)	–	512 (−)	512 (−)	512 (−)	–	256 (−)	–	–
BM47	–	1024 (−)	–	1024 (−)	–	256 (−)	256 (−)	–	512 (−)	512 (−)	–	256 (−)
BM67	256 (−)	512 (−)	–	512 (−)	–	256 (−)	512 (−)	1024 (−)	1024 (−)	–	–	–
BM94	512 (−)	1024 (−)	–	–	–	256 (−)	512 (−)	–	1024 (−)	256 (−)	–	128 (−)
*S. aureus*												Ciprofloxacin
ATCC25923	128 (−)	1024 (−)	–	256 (−)	–	128 (−)	256 (−)	256 (−)	1024 (−)	–	–	1 (8)
MRSA 3	–	–	–	–	–	1024 (−)	1024 (−)	–	1024 (−)	–	–	32 (128)
MRSA 4	256 (1024)	256 (−)	1024 (−)	128 (−)	–	512 (−)	256 (−)	512 (−)	256 (−)	128 (−)	–	64 (128)
MRSA 6	1024 (−)	–	1024 (−)	512 (−)	–	1024 (−)	256 (−)	512 (−)	128 (−)	512 (−)	–	64 (128)
MRSA 8	128 (1024)	1024 (−)	–	256 (−)	–	256 (−)	256 (−)	128 (1024)	1024 (−)	128 (−)	–	16 (64)
MRSA 11	–	256 (−)	–	512 (−)	–	512 (−)	512 (−)	512 (−)	1024 (−)	512 (−)	–	128 (256)
MRSA 12	1024 (−)	–	–	512 (−)	–	512 (−)	128 (−)	512 (−)	1024 (−)	128 (−)	–	32 (32)

RA: reference antibiotics; −: MIC or MBC not detected up to 1024 μg/mL for plant extracts and 256 μg/mL for reference antibiotics; MBC values are in bracket

## Discussion

The emergence of diseases due to MDR bacterial strains is a phenomenon of growing concern worldwide, being qualified by WHO as a “slow-moving tsunami” [7]. Medicinal plants are a promising alternative for the discovery of new anti-infective agents capable to fight against MDR phenotypes; hence, several phytochemicals have been tested against multi-resistant phenotypes [3, 8].

According to Simões et al. [9], a plant extract or phytochemicals can be considered as antimicrobials if the MIC obtain during in vitro tests is in the range 100–1000 μg/mL. The antibacterial activity obtained with *E. prostrata* extract is important as the extract was active on all MDR bacterial strains tested. MIC value of 256 μg/mL was recorded on strains over-expressing efflux pumps AcrAB-TolC (*E. coli* AG102 and *E. aerogenes* CM64) and MexAB-OprM (*P. aeruginosa* PA124) as well as against all MRSA strains. This remarkable activity on Gram-negative as well as Gram-positive bacteria may be due to the presence of phytochemicals that exhibit antimicrobial potential such as quercetin and kaempferol; in fact, their antibacterial properties’ vis-à-vis MRSA and multi-resistant *Propionibacterium acnes* were reported [10, 11]. Moreover, Tala et al. [12] have highlighted the in vivo anti-salmonella potential of this plant. The extracts from leaves of *R. macrophylla* and *M. tomentosa* exhibited better antibacterial activity than those obtained from their barks; this selective activity may be due the qualitative and/or quantitative difference in phytochemical contents of parts of the plants. The antibacterial activity obtained with *R. macrophylla* extracts may be due to the alkaloids present in this plant; in effect, Erasto et al. [13] have demonstrated the anti-mycobacterial extracts properties of alkaloids extracts from *R. macrophylla*. *Viscum album* extracts from various parts of plant were active on all bacterial species used in this study. This reflects the broad spectrum of activity phytochemical compounds available in these extracts like the triterpenes, flavonoids, alkaloids [14]. *Pseudomonas aeruginosa* strains (PA01 and PA124) were not susceptible to the leaves extract of *C. purpurascens* although the fact that this was active on 29 strains out of the 36 tested (including all the MRSA strains); Voukeng et al. [3] have highlighted the involvement of efflux pumps-type RND as the major phenomenon of resistance of Gram-negative bacteria herein studied vis-à-vis of some plant extracts. The susceptibility of the studied MDR bacteria vis-à-vis of *A. floribunda* extracts varied depending on the part of the plant used; these results corroborate those obtained by Siwe et al. [15]. Who got MIC value of 130 μg/mL and 2000 μg/mL with the methanol extracts of the leaves and bark respectively against *S. aureus* ATCC 25923. Okoye and Ebi [16] showed that fractions from leaves extract of *A. floribunda* contained mostly terpenoids, and possessed antibacterial activity against *P. aeruginosa*, *Salmonella keitambii* and *Bacillus subtilis*. Likewise, some flavonoids isolated from this plant such as taxifolin had MIC value of 225 μg/mL on the *S. sobrinus* [17].

## Conclusion

This study highlights the efficacy of some Cameroonian medicinal plants against MDR phenotypes and the results obtained can serve as preliminary test for further experiments to isolate phytochemicals constituents with wide range antibacterial activity.

## Abbreviations

ATCC: American type culture collection
DMSO: dimethyl sulfoxide
*E. prostrata*: *Euphorbia prostrata*
HNC: National Herbarium of Cameroon
INT: *p*-iodonitrotetrazolium chloride ≥ 97% (INT, Sigma-Aldrich)
MBC: minimal bactericidal concentration
MDR: multidrug resistant
MHB: Mueller–Hinton Broth
MIC: minimal inhibitory concentration
RA: reference antibiotic
